# Comparison of the efficacy of tibial transverse transfer and periosteal distraction techniques in the treatment of diabetic foot refractory ulcers

**DOI:** 10.3389/fsurg.2024.1396897

**Published:** 2024-10-03

**Authors:** Yang Yang, Fang Chen, Yiguo Chen, Wei Wang

**Affiliations:** ^1^ Department of Orthopedics, People’s Hospital of Yubei District, Chongqing, China; ^2^ Department of Infectious Disease, People’s Hospital of Yubei District, Chongqing, China

**Keywords:** periosteal distraction, tibial transverse transfer technique, diabetic foot ulcers, distraction osteogenesis, diabetes

## Abstract

**Objective:**

To investigate the efficacy of comparing tibia transverse transport (TTT) and periosteal distraction in treating diabetic foot ulcers.

**Methods:**

A retrospective analysis of 19 patients with diabetic foot ulcers treated with both procedures between February 2020 and November 2022, 8 of whom were treated with the tibial transverse transfer technique (transfer group) and 11 with the osteochondral distraction technique (distraction group), was performed to compare and analyze the clinical efficacy of the two methods.

**Results:**

All wounds were healed in both groups, and the healing time ranged from 15 to 41days with a mean of 28d. The limb preservation rate was 100%. The operative time, intraoperative bleeding, and pain score in the operative area were significantly less in the distraction group than in the removal group, with statistically significant differences (*P* < 0.05). Intra-group comparison between the two groups of patients after surgery revealed that the skin temperature, ABI, TcPO2, SWM and VAS of the affected limb were significantly improved compared with those before surgery, and the difference was statistically significant (*P* < 0.05). The expression levels of VEGF, bFGF, EGF and PDGF were significantly higher than before surgery in both groups, and the difference was statistically significant (*P* < 0.05). No statistically significant differences were found in skin temperature, ABI, TcPO2, SWM, VAS, VEGF, bFGF, EGF and PDGF between the two groups at the corresponding time points preoperatively and postoperatively (*P* > 0.05).

**Conclusions:**

The Periosteal distraction technique can significantly promote the healing of diabetic foot ulcers. It has the same efficacy as TTT in promoting the healing of diabetic foot ulcer wounds and improving the peripheral circulation of affected limbs. In addition, the periosteal distraction technique has the advantages of small trauma, simple operation, few complications, and convenient nursing care.

## Introduction

Diabetic Foot ulcer(DFU)is one of the most severe complications in diabetic patients in late stages, often causing distal ischemia and neuropathy in the affected limb and finally leading to skin necrosis infection and profound tissue destruction (1). Due to the failure to improve the underlying state of ischemia and hypoxia in the affected limb, the recurrence rate is still as high as 40% within one year after healing by medical and surgical debridement (2). In 2001, Qu Long et al. reported for the first time in China the transverse tibial transfer technique based on Professor ILiazrov's “law of tension-stress" (3). This technique can promote microcirculation, nerve reconstruction, and regeneration by continuously and steadily pulling the local bone tissue, thus improving the local microenvironment of the trauma and promoting various growth factors and stem cell production to help tissue regeneration (4–6). Many practical applications have shown that TTT has achieved good efficacy in difficult-to-heal diabetic foot ulcers (7, 8). However, at the same time, its complications, such as secondary fractures at the osteotomy, skin necrosis, and nail tract infections, have caused significant problems for patients (9). Given this, Professor Zeng Naxin derived the lateral tibia periosteum distraction technique and applied it to treat diabetic foot ulcers. The lateral tibia periosteum distraction technique was applied to treat diabetic foot and achieved satisfactory results (10). Moreover, periosteal distraction has the advantages of less trauma, lower cost, and fewer complications, which is more acceptable to patients. However, it is still controversial whether the efficacy of the two procedures is consistent in the diabetic foot, so we retrospectively analyzed the clinical data of 19 patients with diabetic foot treated with the two procedures in our hospital from February 2020 to November 2022. It is reported as follows.

## Materials and methods

### Inclusion criteria

(1) Patients with diabetic foot ulcers Wagner classification (1) of grades 3–4 (infection damage involving deep tissues but not the ankle) have been ineffective for more than two months through dressing changes, debridement,and standard medical treatment; (2) preoperative CT angiography or angiographic ultrasound demonstrating patency of the superficial femoral and popliteal arteries and patency of at least one branch of the anterior tibial, posterior tibial, or peroneal arteries to the plane of the ankle joint (11); (3) good compliance and complete follow-up data.

### Exclusion criteria

(1) Combination of other serious uncontrollable diseases that cannot tolerate the procedure, such as diabetic ketoacidosis, cardiovascular disease, or systemic infection; (2) those with broken and infected skin of the affected lower leg or tibia with an internal fixation device; (3) those who have serious psychiatric disorders and cannot cooperate with the external frame adjustment. [This study was approved by the Medical Ethics Committee of Chongqing Yubei District People's Hospital (No. 2022C1), and all signed an informed consent form before surgery].

### General information

Nineteen patients with diabetic foot ulcers were included in this study. DFU treated with the tibial transverse transfer technique were classified as the transfer group (8 cases), including 5 males and 3 females. The DFU treated with the periosteal distraction technique was classified as the distraction group (11 cases), including 7 males and 4 females. All patients were followed up for approximately 4.8–12 months, with a mean of 8.4 months. No statistically significant differences were found in comparing general information and preoperative wound wanger grading between the two groups (*P* > 0.05) (Tables 1, 2).

**Table 1:** Comparison of general information between two groups of patients (X±s).

Group	*n*	Age (year)	Duration of diabetes (year)	DF process (month)	Albumin (g/L)	BMI (kg/m^2^)
Transfer	8	62.72 ± 10.35	8.59 ± 2.47	3.95 ± 0.64	31.27 ± 6.29	20.77 ± 4.73
Distraction	11	63.49 ± 9.79	7.47 ± 2.23	3.67 ± 0.53	30.98 ± 5.83	21.25 ± 4.26
*t*		0.1653	1.0337	1.0428	0.1036	0.2316
*P*		0.8707	0.3158	0.3116	0.9187	0.8196

**Table 2:** Comparison of wound conditions between two groups of patients.

Group	*n*	Wanger grading		Trauma area		
		3	4	Right foot	Left foot	Bipedal
Transfer	8	4	4	3	5	0
Distraction	11	5	6	4	5	2
*Z*	0.1907			0.5061		
*P*	0.8488			0.6128		

## Treatment method

### Preoperative treatment

(1) If there is no contraindication for all patients to improve the Computed Tomography Angiography (CTA) of the lower limbs after admission, a color Doppler ultrasound of arteriovenous vessels in the lower limbs should be performed to understand the vascular occlusion if the CTA cannot be improved. (2) The diabetic diet plan was formulated jointly with the Endocrinology Department before the operation, and the joint ward round was conducted at least once a week to regulate blood glucose. The blood glucose control standards were as follows: 2 h after meals, blood glucose ≤10.0 mmol/L, fasting blood glucose ≦8 mmol/L, and albumin level ≥30 g/L. (3) Culture the wound secretions, and the Joint Infection Department selected antibiotics according to the culture and drug sensitivity results and dynamically followed up to adjust the medication.

### Surgery method

The transfer group: after satisfactory anesthesia with lower limb nerve block without a tourniquet, routine disinfection, and towel laying. A 3 cm-long curved incision was made on the medial side of the tibial tuberosity about 5 cm distal to the periosteum (without cutting the periosteum) to determine the extent of the transverse tibial bone transfer and osteotomy (5.0 cm in length and 1.5 cm in width). A 2.5 mm drill was used as a guide to making successive holes at 2 cm intervals within the predetermined bone window, and a 4 mm stainless steel half-pin was screwed in to transfer the bone. Then, a bone knife was used to pry the displaced bone block along the drilled hole to move the bone window up and down. A 0.5 mm external fixation stainless steel half-pin was screwed into each of the proximal and distal tibias at about 1 cm of the bone window (penetrating both layers of the cortex). The bone transfer device was assembled and fixed firmly, and the subcutaneous tissue and skin were sutured. After surgery, the distal diabetic foot wound was cleared, and Vacuum Sealing Drainage(VSD) negative pressure suction was placed to promote wound healing according to the condition of the wound. On the third postoperative day, the external fixation brace was adjusted for bone transfer, and the bone block was first moved outward at a rate of 0.5 mm/24 h. After moving for 14 days, the bone was left standing for three days, and then moved back at the same speed. After the transfer, the external brace was fixed for 6–8 weeks until the bone window healed and the external brace was removed.

The distraction group: after satisfactory anesthesia with lower limb nerve block without a tourniquet, routine disinfection, and towel laying. A longitudinal incision of approximately 1 cm was made on the anterior medial side of the proximal calf (4 cm below the tibial tuberosity and 1 cm medial to the tibial spine), and the tissue was separated to reach the periosteum. The periosteum is incised transversely with a sharp knife for approximately 1 cm. Then, the periosteum is gently dissected up and down along the tibial stem with a special periosteal stripper to the length of the pre-inserted plate, avoiding excessive dissection that may result in insufficient postoperative retraction. Then, four quadrants were established with the skin and periosteal incision as the longitudinal and transverse axes. A 2.0 mm drill was used to penetrate the unilateral bone cortex at each of the four quadrants to perform medullary decompression, followed by the insertion of a 0.8cm × 0.8 cm distraction plate at the periosteal incision, all of which were inserted distally and moved in the opposite direction until the midpoint of the plate was confirmed to be at the periosteal incision and the midline of the upper and lower margins of the tibial stem. A flat-headed hollow screw was screwed into the middle of the plate, followed by a 1.5 mm kerf pin in the hollow screw hole and penetrating the contralateral bone cortex to fix the plate and the hollow screw, and after satisfactory fixation, C-arm fluoroscopy ensured that the distraction plate was placed along the midline of the anterior and posterior margins of the tibial stem and that the screw and kerf pin was well positioned. Finally, the periosteum was observed to lift with the screw plate by twisting the caudal cap of the hollow screw clockwise. The cremaster's pin was cut parallel to the caudal cap of the screw so that the caudal end was just over the retractor screw by approximately 0.2 cm. The periosteum was closed with 3-0 absorbable sutures, and the incision was fully closed. A distal diabetic foot debridement was then performed. The retraction of the periosteum was started on postoperative day 3, with a clockwise retraction rate of 0.25 mm/12 h per day, and the retraction screws and plates were removed after 20d of continuous retraction. The patient was allowed to leave the bed for daily activities during the retraction period. The dressing of periosteal stretch wound was changed every two days, and the external nail canal was disinfected with 75% alcohol every day to prevent nail canal infection. (The surgical tools of Zhejiang Kehui Medical Devices Co.).

### Clinical observation indexes

Before operation, 14 days, 28 days and 90 days after move, the pain score (NRS) method (from 0 to 10, patients are required to choose a value representing their pain, 0 means no pain, the higher the value, the greater the pain) was used to record the operation time, blood loss and pain sensation in the operation area of the two groups. Then skin temperature, ankle-brachial index (ABI) and percutaneous oxygen partial pressure (TcPO_2_ > 40 mmHg: no bloody lesions; 21 mmHg–39 mmHg: mild ischemic lesion; TcPO_2_ < 20 mmHg: severe ischemic lesion (12) to evaluate the peripheral circulation of the affected limb. Semmes-Weinstein Monofilament (SWM) is used to evaluate the improvement of neuropathy around diabetic foot ulcer (covering patients’ eyes, placing monofilaments with different specifications perpendicular to the affected foot, and uniformly exerting force to bend the monofilaments to 90 degrees, and the position that can be correctly perceived twice in three consecutive times is “+”. Normal specification (1.65–2.83), mild tactile hypoesthesia (3.22–3.61), protective sensory hypoesthesia (3.84–4.31), protective sensory loss (4.56–6.65) and total sensory loss (specification >6.65) (13).The progress of pain in the affected limb was evaluated by visual analogue scale (VAS). Finally, the healing time of diabetic foot wound, limb preservation rate and complications (nail infection, displaced site fracture and skin necrosis in operation area) were observed and recorded in the two groups.

### Laboratory observation indexes

5 ml of fasting peripheral venous blood was drawn before surgery and at 7:00 am on the 7 days, 21 days, and 35 days after the start of the move and placed upright for a moment to allow the whole blood to clot; the serum and blood cells were separated by centrifugation at a radius of 10 cm and 2,000 r/min for 10 min, and 1.5 ml of the supernatant was placed in a centrifuge tube and stored at −80℃ in a refrigerator. The levels of VEGF, bFGF, EGF and PDGF were determined by enzyme immunoassay kits (provided by Hangzhou Biotechnology Co, Ltd.) using vascular endothelial growth factor (VEGF), essential fibroblast growth factor (FGF), epidermal growth factor (EGF) and platelet-derived growth factor (PDGF).

### Statistical methods

SPSS 23.0 statistical software was used for statistical analysis, and the measurement data were expressed as (X±s), and *t*-test for independent samples was used for comparison between two groups, and a variance test was used for comparison between three groups. The rank sum test was used for the comparison of hierarchical data. All test levels *α* = 0.05.

## Results

### Comparison of clinical observation indexes

**Figure 1:**
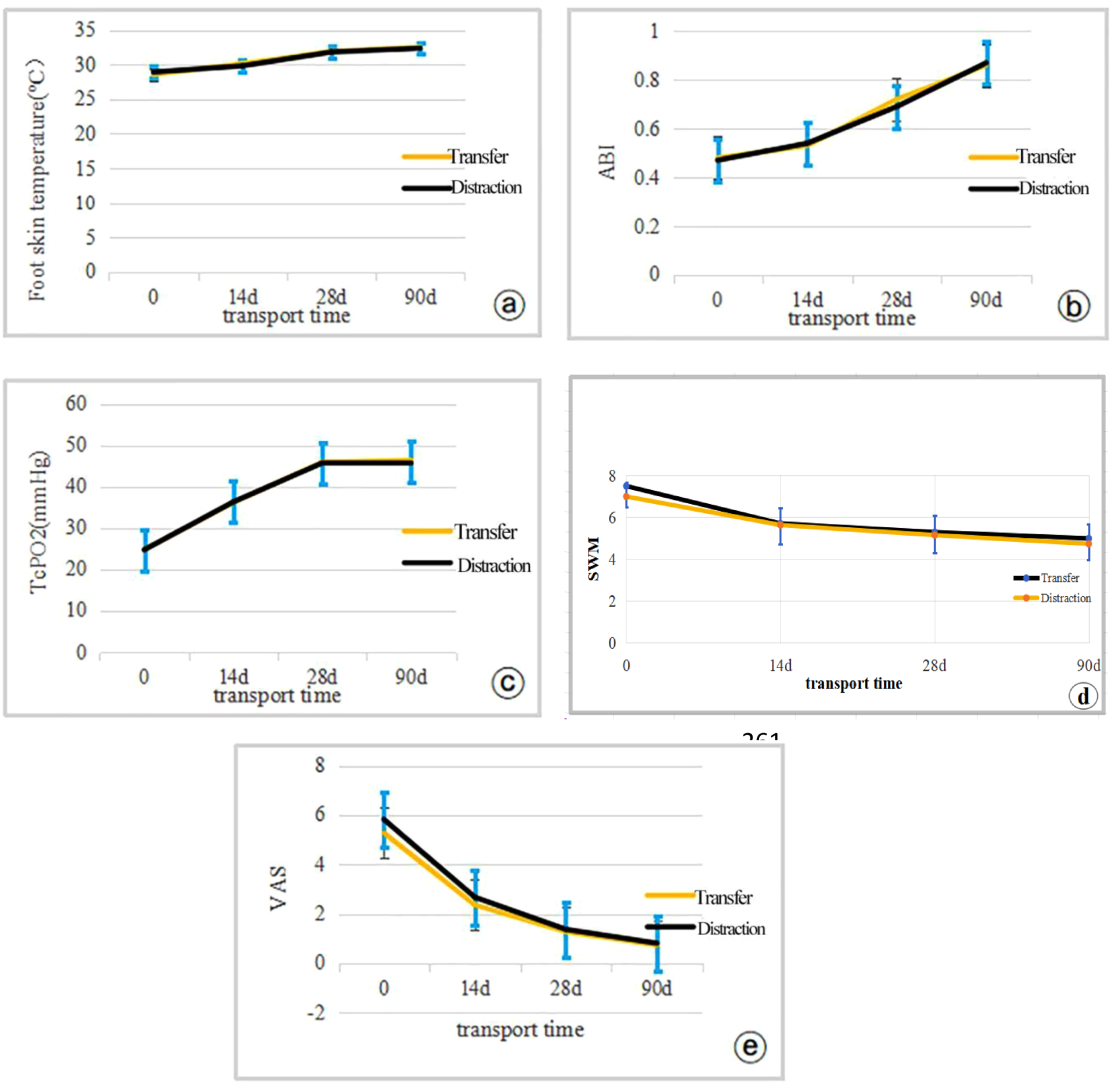
Expression levels of clinical efficacy observations were compared between the two groups of patients using independent samples *t*-tests (0d indicates preoperative **(a)** temperature; **(b)** ABI; **(c)** TcPO_2_; **(d)** SWM; **(e)** VAS.

**Figure 2:**
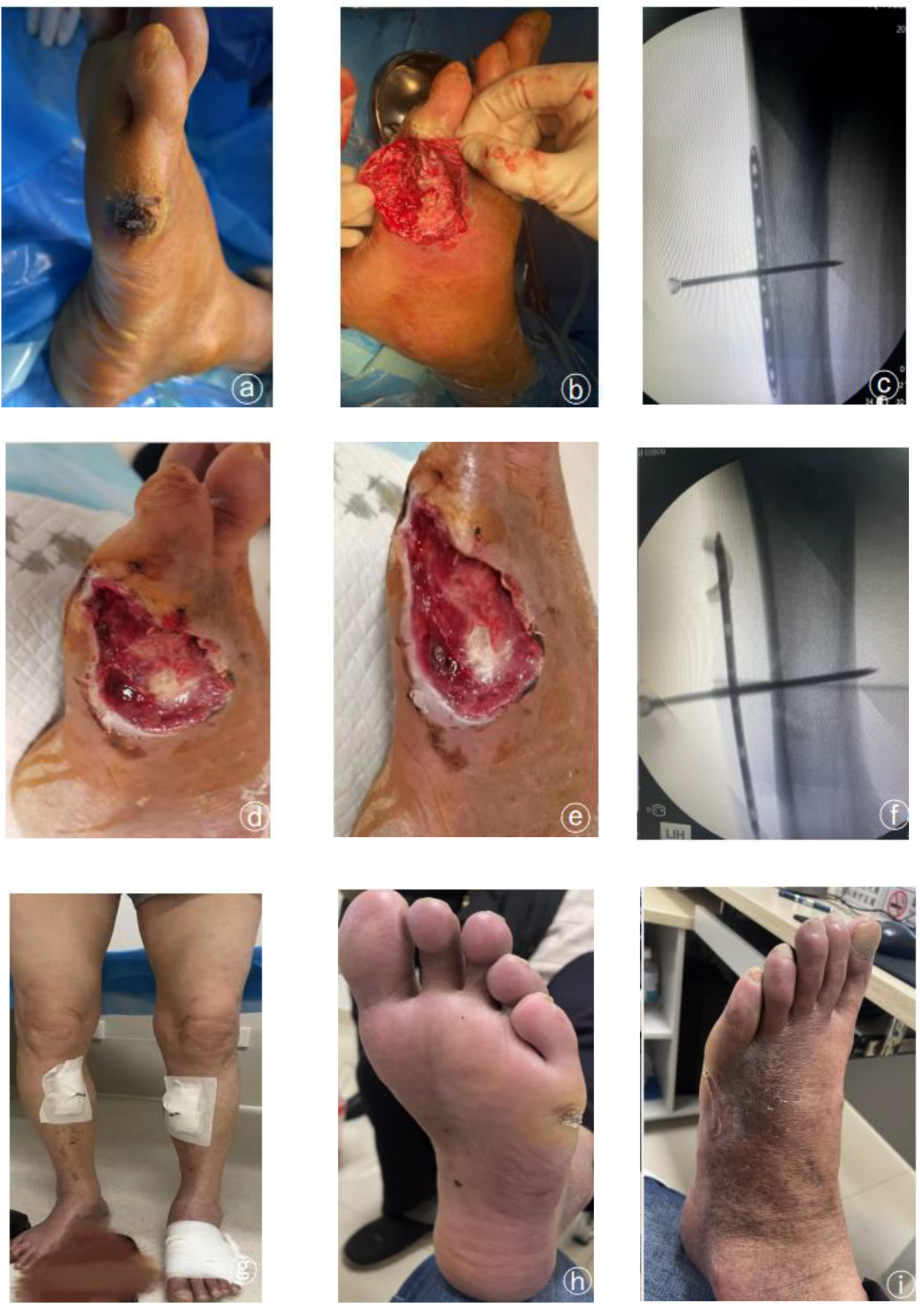
Typical case patient, male, 67 years old, left diabetic foot wanger grade 4, double lower limb arterial occlusion stenting after stenting with HIV, requesting limb preservation treatment. **(a)** preoperative trauma; **(b**,**c)** periosteal distraction and local debridement; **(d**,**e)** large amount of fresh granulation tissue growth on the trauma was seen after 20 days of distraction; **(f)** the plate was lifted away from the bone surface after 20 days of distraction; **(g)** the patient could go down to the ground normally after bilateral periosteal distraction; **(h,i)** local trauma was completely healed after 12 weeks of the operation.

The operative time, intraoperative bleeding and pain score in the operative area were significantly less in the distraction group than in the transfer group, with statistically significant differences (*P* < 0.05). The intra-group comparison between the two groups revealed that the skin temperature ABI, TcPO2, SWM and VAS of the affected limb were significantly improved compared with those before surgery, and the difference was statistically significant (*P* < 0.05). No statistically significant differences were found in skin temperature, ankle-brachial index, TcPO2, SWM and VAS between the two groups of patients compared at the corresponding time points preoperatively and postoperatively (*P* > 0.05). All wounds were healed in both groups, with healing times ranging from 15 to 41 days, with a mean value of 28d. The limb preservation rate was 100%. There were two complications in the transfer group: One patient in the displaced group had a fracture in the osteotomy area, and a nail tract infection accompanied one patient. All patients in the distraction group were successfully discharged from the hospital with the external frame support removed. (Tables 3, 4; Figures 1, 2).

**Table 3:** Comparison of surgical data between the two groups (X±s).

Group	Operation time (min)	Intraoperative bleeding (ml)	NRS
Transfer	63.64 ± 8.92	47.93 ± 5.56	6.74 ± 0.49
Distraction	21.79 ± 4.26	12.52 ± 1.28	3.58 ± 0.24
*t*	13.6655	20.5941	18.6655
*P*	0.0000	0.0000	0.0000

**Table 4:** Comparison of clinical observation indexes between two groups (X±s).

Group		Preoperative	14 days after move	28 days after move	90 days after move	*F*	*P*
Transfer	Piven (℃)	28.59 ± 0.92	30.16 ± 0.74	31.94 ± 0.95	32.56 ± 0.87	43.6871	0.0000
Distraction		28.94 ± 1.25	29.83 ± 0.96	31.84 ± 0.79	32.39 ± 1.13	28.1056	0.0000
*t*		0.6690	0.8106	0.2504	0.3549		
*P*		0.5125	0.4288	0.8503	0.7270		
Trsnsfer	ABI	0.48 ± 0.12	0.53 ± 0.13	0.72 ± 0.09	0.86 ± 0.07	32.3504	0.0000
Distraction		0.47 ± 0.13	0.54 ± 0.15	0.69 ± 0.07	0.87 ± 0.08	46.9645	0.0000
*t*		0.1708	0.1514	0.8188	0.2830		
*P*		0.8664	0.8814	0.4242	0.7806		
Transfer	TcPO_2_ (mmHg)	24.73 ± 1.21	36.15 ± 1.76	45.89 ± 1.24	46.39 ± 1.37	751.9790	0.0000
Distraction		24.72 ± 1.17	36.28 ± 1.16	45.69 ± 1.34	45.98 ± 1.28	1,021.2516	0.0000
*t*		0.0181	0.1946	0.3312	0.6696		
*P*		0.9857	0.8480	0.7446	0.5121		
Transfer	SWM	7.48 ± 0.83	5.69 ± 0.77	5.28 ± 0.86	4.98 ± 0.97	17.9209	0.0000
Distraction		6.98 ± 0.67	5.62 ± 0.82	5.14 ± 0.93	4.72 ± 0.95	21.1086	0.0000
*t*		1.4540	0.1884	0.3341	0.5839		
*P*		0.1642	0.8528	0.7424	0.5670		
Transfer	VAS	5.27 ± 0.83	2.36 ± 0.42	1.27 ± 0.31	0.73 ± 0.12	184.6545	0.0000
Distraction		5.82 ± 0.94	2.67 ± 0.59	1.37 ± 0.32	0.81 ± 0.13	247.9700	0.0000
*t*		1.3205	1.2667	0.6812	1.3667		
*P*		0.2042	0.2223	0.50490	0.1895		

### Comparison of laboratory observed indicators

**Figure 3:**
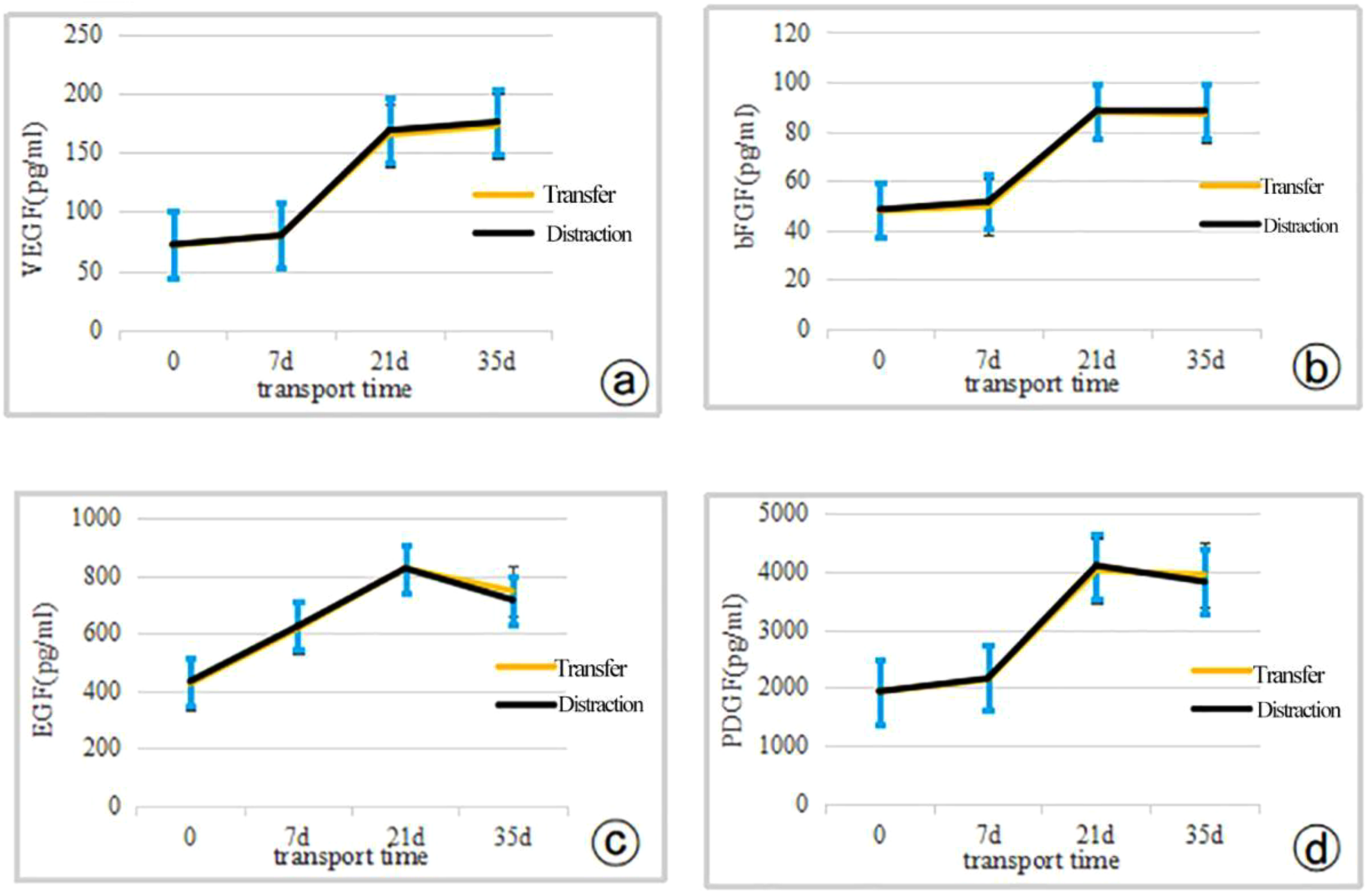
The expression levels of cytokine indicators were compared between the two groups of patients using the independent samples t-test (0d indicates preoperative) **(a)** VEGF; **(b)** bFGF; **(c)** EG F; **(d)** PDGF.

The expression levels of VEGF, bFGF, EGF and PDGF were significantly higher in both groups compared with the preoperative levels, and the differences were statistically significant (*P* < 0.05). There was no statistically significant difference in the expression levels of VEGF, bFGF, EGF and PDGF between the two groups at the corresponding time points before and after surgery (*P* > 0.05). Each index increased with the increase of retraction intensity. The increasing trend was delayed after removing the retraction outer frame in both groups at 35 days after the move, and the levels of EGF, bFGF and PDGF decreased compared with those at 21 days after the move. (Table 5; Figure 3).

**Table 5:** Comparison of laboratory observation indexes between two groups (X±s).

Group		Preoperative	7 days after move	21 days after move	35 days after move	*F*	*P*
Transfer	VEGF	71.59 ± 10.29	79.85 ± 9.74	164.94 ± 35.95	172.56 ± 38.87	26.0793	0.0000
Distraction		72.27 ± 11.28	79.93 ± 10.34	168.75 ± 39.87	175.88 ± 40.42	32.9844	0.0000
*t*		0.1345	0.0020	0.2141	0.1796		
*P*		0.8946	0.9984	0.8330	0.8596		
Transfer	bFGF	47.61 ± 10.82	49.53 ± 15.13	87.72 ± 23.09	86.86 ± 21.07	11.5160	0.0004
Distraction		48.27 ± 11.03	51.43 ± 14.85	88.29 ± 24.15	88.13 ± 22.35	14.5694	0.0000
*t*		0.1298	0.2732	0.0517	0.1252		
*P*		0.8983	0.7880	0.9594	0.9018		
Transfer	EGF	424.73 ± 153.21	616.75 ± 207.48	825.89 ± 286.31	746.54 ± 248.95	6.4695	0.0065
Distraction		434.13 ± 157.31	625.34 ± 209.26	825.77 ± 290.13	715.73 ± 231.25	8.3559	0.0013
*t*		0.1300	0.0887	0.0009	0.2778		
*P*		0.8981	0.9304	0.9993	0.7845		
Transfer	PDGF	1,932.87 ± 875.59	2,134.28 ± 947.86	4,017.69 ± 997.77	3,948.48 ± 969.83	12.4523	0.0003
Distraction		1,934.72 ± 864.95	2,158.54 ± 953.64	4,105.53 ± 991.85	3,823.87 ± 938.82	17.5960	0.0000
*t*		0.0046	0.0549	0.1901	0.2818		
*P*		0.9964	0.9569	0.8515	0.7815		

## Discussion

Hyperglycemia causes calcification, stenosis and even occlusion of peripheral arterioles; Hyperglycemia also leads to the acceleration of oxidative stress in nerve cells, leading to the glycosylation of protein in nerve cells, causing abnormal sensation and decrease of muscle pumps in the affected limbs, stimulating vasoconstriction, thus aggravating the distal ischemia of the affected limbs and eventually leading to skin ulcers and infections. Hyperglycemia and peripheral neuropathy, as two complementary and independent risk factors, are one of the main reasons to promote the progress of diabetic foot ulcer (14). The cyclic development of vascular occlusion, neuropathy, and local infection severely restricts the treatment of diabetic foot. Secondly, during the wound healing process, the demand for oxygen and nutrients is high due to a large number of capillary neovascularization and cell proliferation, migration, and metabolic activities (15). So, the primary goal of diabetic foot treatment is to rebuild the peripheral circulation of the affected limb, break its cyclic reciprocal pathological state, and bring nutrients for tissue repair. Previously, the improvement of diabetic foot circulation could only rebuild the blood flow in the large arteries but not the microcirculation, and the root cause of the difficulty in healing diabetic foot ulcers, namely small artery occlusion and microperfusion damage in the foot, has not been addressed (16). Even if diabetic foot ulcers are made to heal by conventional treatment, their 1-year recurrence rate is as high as 40%, and the 5-year recurrence rate is as high as 65% (2). The amputation rate of diabetic foot patients with Wanger grade 3 and above is as high as 90%, and the mortality rate five years after amputation is as high as 70% (17). Because of this, the lateral tibial bone transfer technique based on Professor ILiazrov's theory of “*in situ* tissue regeneration and natural repair and reconstruction” has become a powerful treatment for difficult-to-heal diabetic foot wounds with the pioneering improvement of Chinese scholars Qikai H and Sihe Q (8). Its continuous and stable tension on the unilateral bone and accessory tissues can activate the body's natural repair potential and promote microvascular and nerve regeneration in the affected limb (18). Some scholars have named the phenomenon of “summoning” as the final cure for persistent foot diseases by performing distraction surgery away from gangrenous and ulcerated infected areas (11).

**Figure 4:**
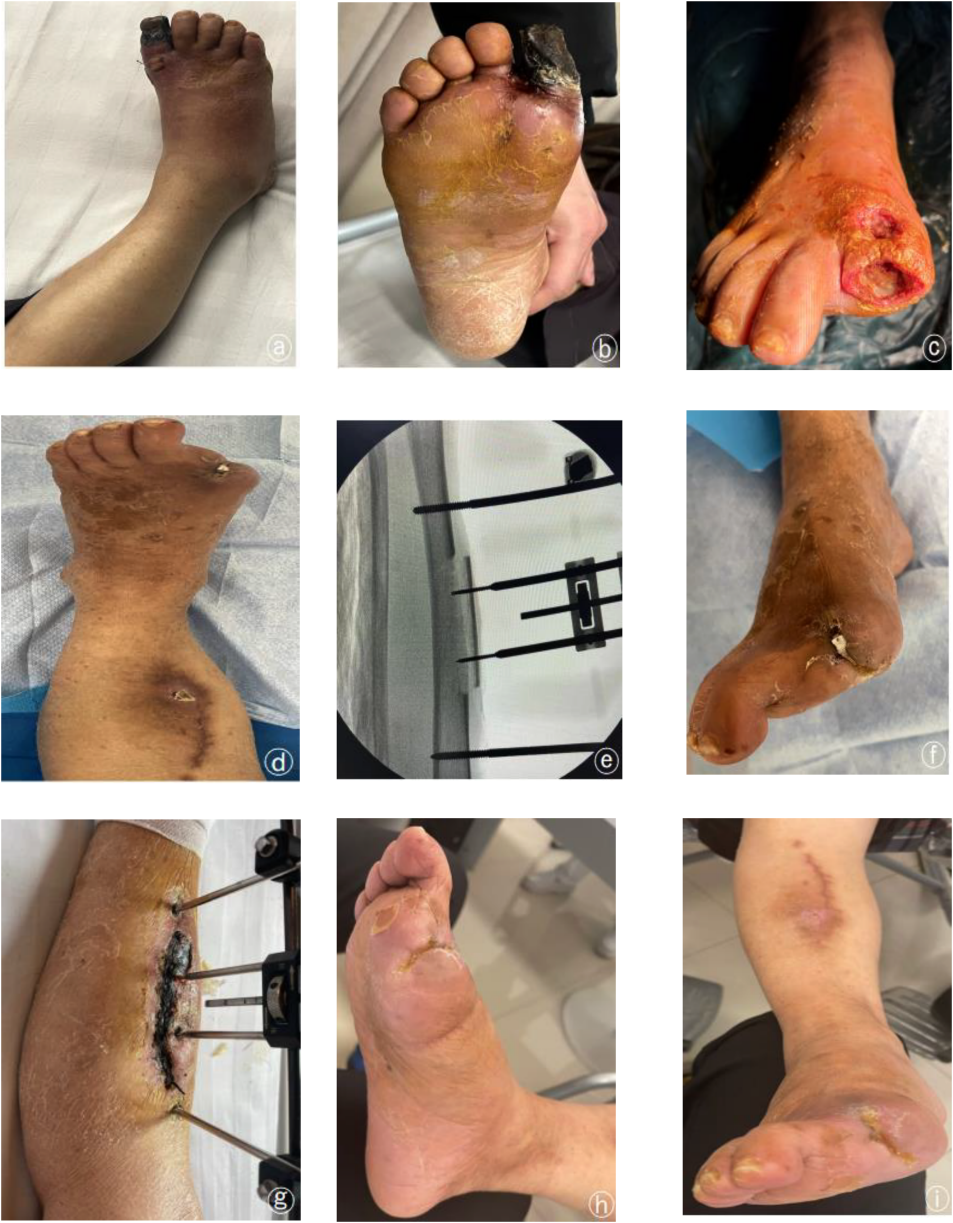
Typical case patient, female, 65 years old, right diabetic foot wanger grade 4, severe occlusion of arteries in both lower limbs, requesting limb-sparing treatment. **(a,b)** Preoperative trauma, blackened necrosis of the lesser toe is seen; **(c)** Localized trauma after clearing of the distal diabetic foot during lateral tibial bone transfer. **(d**–**f)** Basic healing of the trauma was seen after removal of the bone transfer external fixation frame at 6 weeks postoperatively, but the local field was not aligned; **(g)** necrosis of the skin flap in the surgical area; **(h**,**i)** complete healing of the trauma at 20 weeks after TTT.

The clinical application of the TTT technique has achieved good efficacy in treating refractory wounds of the lower extremities (8, 11). Studies have shown that the healing and limb preservation rates of Wagner grade 3 diabetic foot ulcer wounds treated by the TTT technique exceeded 95%, and the 1-year recurrence rate was less than 10% (19). Our study showed significant improvement in foot skin temperature, ABI, TcPO2, SWM and VAS in the bone relocation group compared to the preoperative period, with successful healing of all ulcer wounds and 100% limb preservation rate. However, complications accompanying the TTT technique, such as fracture at the osteotomy, skin necrosis in the osteotomy area, nail tract infection, and deformity thickening of the osteotomy segment, hindered the further promotion of this technique (9) (Figure 4). As the research of the discipline advanced, the study of FU et al. found that similar to distraction osteogenesis, there was also abundant capillary proliferation in the distraction of the periosteum, which they attributed to the rich undifferentiated vascular, vascular, and neural network in the periosteum, which could also promote the regeneration of vascular nerves and have the osteogenic ability when it was subjected to continuous distraction (20). In 2019, professor Naxin Z et al. first applied the periosteal distraction technique to treating diabetic foot and achieved good efficacy (21). Subsequently, some scholars reported that the effect of the periosteal distraction technique was comparable to the TTT technique in the treatment of diabetic foot in Wagner grade 2–3. At the same time, there are different views on the treatment effect of patients in Wanger grade 3–4 (22). The results of this study suggest that the TTT technique and the osteochondral distraction technique are equally effective in treating diabetic foot ulcers of Wanger grade 3–4, and the patient's symptoms were significantly improved compared with those before surgery. There was no statistical difference during the follow-up (*P* > 0.05). At the same time, the operating time and bleeding volume of the distraction group were much less than those of the removal group. The external fixation frame of the distraction group was more aesthetically pleasing and lighter than the removal group. The pain in the operated area was much less than that of the removal group due to the osteotomy and postoperative fixation of multiple segments. These advantages make distraction surgery more acceptable for diabetic foot ulcer patients and significantly reduce the stress of patient care. Finally, Dingwei Z et al. concluded that the TTT technique has a much higher incidence of secondary osteotomy fractures and nail tract infections than periosteal distraction (9), consistent with our statistical analysis of complications in both groups. This shows that the osteochondral distraction technique's superiority over the TTT technique in terms of surgical operation and postoperative care deserves recognition.

The TTT technique can modulate local and systemic inflammatory responses, and the slow and continuous tension stress not only promotes capillary proliferation on the affected side but also stimulates regeneration of the contralateral capillary network (23). Wound healing requires granulation tissue filling, and granulation tissue production requires abundant blood vessels and a large amount of cell growth factors (24). Xu et al. showed that continuous and steady low-stress retraction can modulate the production of HIF-1/vascular endothelial growth factor (VEGF), promoting neovascularization and maintaining the normal state and integrity of blood vessels. At the same time, the local application of VEGF can vascularize wounds and enhance wound healing (25). bFGF, a chemokine for endothelial cell proliferation, can directly promote endothelial cell proliferation and regulate VEGF secretion by endothelial cells to induce neovascularization (26). EGF stimulates the migration of epidermal cells and fibroblasts to the damaged site in wound repair, promotes collagen production, protein synthesis, DNA and RNA repair, and granulation tissue formation by fibroblasts (27). PDGF encourages the migration of neutrophils and macrophages to the damaged site to play a clearing role and promotes the secretion of new extracellular matrix by fibroblasts and IGF-1 mediated re-epithelialization (28). All these cytokines play essential and complementary roles in the repair of wounds. Current studies have shown that slow and sustained low mechanical tension stress can promote the expression of VEGF, bFGF, EGF, and PDGF, which may be one of the mechanisms of action to encourage diabetic foot wound healing (29, 30). In our study, we found that the levels of VEGF, bFGF, EGF and PDGF increased significantly with the continuation of retraction compared with the preoperative period, but when the retraction support was removed bFGF, EGF and PDGF decreased slightly compared with the previous follow-up.

In contrast, VEGF increased somewhat compared with the previous period. Accordingly, we suggest that continuous low-stress distraction of bone tissue and its accessory findings can promote the expression of cell growth factors such as VEGF, bFGF, EGF and PDGF. In addition, Wei Gao et al. found that continuous retraction also promoted the production of M2 macrophages, which can inhibit the local inflammatory response and reconstruct the damaged polarization balance of macrophages, thus promoting wound healing (31).

In conclusion, the TTT technique under the “tension-stress rule” can promote wound healing. In our study, we found that the TTT and the periosteal distraction techniques have the same efficacy in promoting the healing of Wanger grade 3–4 diabetic foot ulcers and improving the peripheral circulation of the affected limbs. The osteophyte distraction technique has the advantages of less trauma, more straightforward operation, fewer complications, and more accessible care. However, the efficacy of the osteotomy retraction technique on heavy Wagner grade 5 diabetic foot ulcers still needs more validation because osteotomy decompression and osteotomy retraction operations were not performed on the tibia. The present study has limitations due to the small number of cases included, and future multicenter, large sample, and high-quality studies are needed to investigate the efficacy mechanism of the periosteal distraction technique. Finally, it is hoped that periosteal retraction will provide a new option for the surgical treatment of diabetic foot ulcers.

## Data Availability

The original contributions presented in the study are included in the article/Supplementary Material, further inquiries can be directed to the corresponding author.
